# Enhanced Single Shot Small Object Detector for Aerial Imagery Using Super-Resolution, Feature Fusion and Deconvolution

**DOI:** 10.3390/s22124339

**Published:** 2022-06-08

**Authors:** Mahdi Maktab Dar Oghaz, Manzoor Razaak, Paolo Remagnino

**Affiliations:** 1 Faculty of Science and Engineering, Anglia Ruskin University, Cambridge CB1 1PT, UK; 2 The Robot Vision Team, Kingston University London, London KT1 2EE, UK; manzoor.razak@kingston.ac.uk; 3 Department of Computer Science, Durham University, Upper MountJoy, Durham DH1 3LE, UK; paolo.remagnino@gmail.com

**Keywords:** deconvolution, feature fusion, small object detection, SSD, super-resolution

## Abstract

One common issue of object detection in aerial imagery is the small size of objects in proportion to the overall image size. This is mainly caused by high camera altitude and wide-angle lenses that are commonly used in drones aimed to maximize the coverage. State-of-the-art general purpose object detector tend to under-perform and struggle with small object detection due to loss of spatial features and weak feature representation of the small objects and sheer imbalance between objects and the background. This paper aims to address small object detection in aerial imagery by offering a Convolutional Neural Network (CNN) model that utilizes the Single Shot multi-box Detector (SSD) as the baseline network and extends its small object detection performance with feature enhancement modules including super-resolution, deconvolution and feature fusion. These modules are collectively aimed at improving the feature representation of small objects at the prediction layer. The performance of the proposed model is evaluated using three datasets including two aerial images datasets that mainly consist of small objects. The proposed model is compared with the state-of-the-art small object detectors. Experiment results demonstrate improvements in the mean Absolute Precision (mAP) and Recall values in comparison to the state-of-the-art small object detectors that investigated in this study.

## 1. Introduction

Object detection is one of the core research areas in computer vision. Recent breakthroughs in Convolutional Neural Network (CNN) and object detection unlocked new horizons and possibilities in various domains ranging from security and surveillance applications, such as face detection, crowd analysis and activity recognition to medical image analysis and self-driving vehicles research [1,2,3,4].

**Figure 1:**
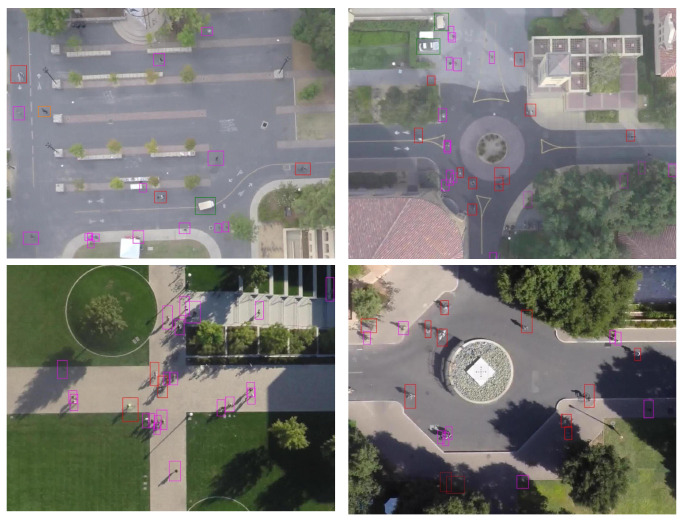
Objects in UAV images are usually small in size (proportional to total image size) and general purpose object detectors are not designed to cope with it [11].

Despite the contextual similarities of these domains, they utilize different image acquisition techniques that often require significant adaptation and alteration of the state-of-the-art general purpose object detectors to achieve desirable results. A prominent example of such a domain is Unmanned Aerial Vehicles (UAV) imagery. The UAV imagery is getting more popular than ever before with a variety of applications including smart farming [5], search and rescue [6], disaster management [7], archaeological structure modeling [8], security and surveillance [9] and many others. In UAV imagery, due to the flight altitude, the top-down camera perspective and wide-angle lenses, object shapes and appearances are relatively unconventional and they usually take up a small fraction of the image area, as illustrated in Figure 1. General purpose object detectors are trained and tuned on datasets, such as ImageNet and COCO, which mainly offer ground-level medium-sized images. These detectors fail to provide good detection accuracy when it comes to out-of-ordinary small objects captured by UAVs. A reliable small object detection demands mechanisms that preserve and enhance small object feature representation in the detection layer [10].

Pre-deep learning object detection techniques, such as boosted cascade [12], Histograms of Oriented Gradients (HOG) [13] and Deformable Part Models (DPM) [14] were relatively inaccurate and unreliable for real-world applications; however, availability of GPU computing and abundant of labeled training data (ImageNet) fast-tracked the rise of CNN-based object detection. The CNN-based object detectors have become the preferred choice for many researchers due to their unprecedented accuracy and availability of ample training data and processing power. These approaches can be categorized into one-stage and two-stage object detectors.

The *one-stage* object detectors require only a single pass through the neural network to detect and localize the objects. These methods treat object detection as a simple regression problem by taking an input image and learning the class probabilities and bounding box coordinates. For instance, *You Only Look Once* (YOLO), which is one of the notable *one-stage* object detectors, splits the input image into a grid of *S × S* cells [15]. If a bounding box center falls into a cell, that cell is “responsible” for detecting the existence of that object. More precisely, each cell is in charge of predicting the exact coordinates of bounding boxes, a confidence score indicates the likelihood that the cell contains an object, and a probability of object class conditioned on the existence of an object in the bounding box. The YOLO utilize a fairly standard CNN (similar to GoogLeNet) that receives the input image, extracts spatial features, and at the end outputs an encoded vector designed to predict bounding boxes, confidence for those boxes, and class probabilities.

When it comes to the small objects, the efficiency and reliability of this and similar approaches degrade. To successfully detect small objects, a considerably finer *S × S* grid is required to reliably predict the coordinates of the bounding boxes and maintain the significance of the confidence scores. This exponentially increases the computational complexity of the detector for an insignificant accuracy boost in return. Overall, the *one-stage* object detectors are simpler and faster, although they can sometimes struggle with localization and detection accuracy. The most prominent examples of one-stage object detectors are YOLO and *Single Shot multi-box Detector* (SSD) [16].

On the other hand, *two-stage* object detectors first attempt to extract candidate regions of objects (region proposal), which significantly reduces the number of locations that are likely to contain the objects and then they employ a combination of ConvNets and other techniques to classify and refine the extracted region proposals. For instance, the *Region-based Convolutional Neural Network* (R-CNN) [17], which is one of the earliest *two-stage* object detectors, uses selective search to extract 2000 region proposals. This method employs a graph-based segmentation algorithm [18] to generate an over-segmented segmentation map and then iteratively and hierarchically merges these segments into larger region proposals based on their color, texture, size, and shape similarity.

In the second stage, pre-trained AlexNet is used to extract the feature vector of the cropped/reshaped region proposals. Then, *Support Vector Machines* (SVMs) are used to generate a confidence score classify these regions into different classes. A greedy non-maximum suppression algorithm only retains overlapping regions with a higher confidence scores. Finally, a linear regression model is used to further refine the bounding boxes for each identified object. Two-stage detectors have higher localization and object recognition accuracy; however, these techniques tend to be significantly slower than their *one-stage* counterparts. These methods are fairly slow to begin with and it can be argued that they require a significantly higher number of region proposals to deal with small objects. Moreover, we have realized that the segment merging process is less effective when it comes to small-sized objects. Popular *two-stage* detectors are the R-CNN detectors and the various extensions of it [17,19,20].

In recent years, novel techniques, including Detection Transformer (DETR) [21], Saliency detection [22], Swin Transformer [23], and Hybrid Task Cascade (HTC) [24], have been conceived to improve object detection and segmentation accuracy; while these techniques managed to successfully improve object detection accuracy, their primary focus is on general and reasonably sized objects on datasets such as COCO and not small objects in aerial images.

**Figure 2:**
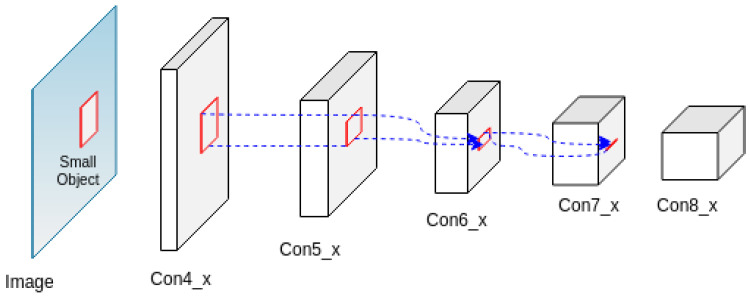
Poor feature representation of small objects at deeper layers of typical Convolutional Neural Networks, which are usually caused by multiple pooling and stride >1 processes.

Most object detection algorithms perform well when the objects are represented with reasonable size, proportion, and resolution; however, when it comes to small-sized objects, they under-perform severely. This is mainly caused by weak feature representation of the small-sized objects in deeper layers of CNN and a significant imbalance between the background and target objects proportion. Though there is no standard definition of the scale of an object to be qualified as a *small object*, the (D)etection, (O)bservation, (R)ecognition and (I)dentification (DORI) criteria conceived by [25] states that 10% of the image height is required for the object observation and detection. In Figure 1 in [11], it can be observed that the objects are considerably small/insignificant proportional to the total image size and can be categorized as small objects. Figure 2 illustrates how a typical CNN fails to properly resolve and represent small objects features. At each layer of the network, features of the small object down-sampled through pooling or stride >1 (typically, with half the resolution size of the previous layer) results in a progressive reduction and sometimes disappearance of small object feature representation at the prediction layer, which deteriorates the learning and detection process.

The majority of the image and video acquisition applications, such as surveillance, autonomous driving, and satellite and aerial imagery, can only capture distant objects in small sizes, mainly due to technological and physical constraints and limitations. Current general purpose object detectors, including SSD, YOLO, R-CNN, CenterNet++, and their variants, under-perform when it comes to the detection of small objects [26]. For instance, although the SSD utilizes a multi-scale feature representation that supposedly provides a relatively better detection accuracy for small objects, it uses a fairly deep CNN (VGG16) in its back-end, which degrades the spatial resolution of the small objects and makes detection of these objects difficult [27,28,29]. CenterNet++ [30], a state-of-the-art object detector that benefits local perception swin transformer in its backbone is capable of generating accurately positioned bonding boxes and achieve outstanding mAP, but it relatively under-performs when it comes to datasets that mainly exhibit small objects.

Therefore, innovative measures are required to develop object detectors, capable to demonstrate high accuracy in detecting small objects. Small object detection is a relevant research problem and is gaining research attention. One approach that is particularly explored for small object detection is the multi-scale feature representations structure in the network architectures. Object detectors such as SSD and Deconvolutional Single Shot Detector (DSSD) [31] use multi-scale representations in their network for predictions. Features from different layers in the network are explored and combined for better representation of object features and to improve the detection of smaller objects. Table 1 provides an overview of some of the recent works related to CNN-based small object detection and summarizes their approaches.

This study attempts to address the small object detection problem using a multi-scale feature representation CNN model. The proposed model design includes the SSD network as the baseline network and extends it with an additional deconvolution module, super-resolution module and a shallow layer feature-fusion module. These three additions result in better preservation of small object features at deeper CNN layers that subsequently improved small objects detection accuracy compared to the base SSD model.

The main contributions of this study are as follows:

- A novel deep model capable of improving feature representation of small objects at the prediction layer that leads to overall better small objects detection accuracy;
- A deconvolution module that up-scales small objects’ feature resolution and provides more details to the prediction layer;
- A super-resolution module that applies residual and up-sampling blocks to shallow layers and improves scale invariancy and enhances resolution of the small objects at the prediction layer;
- A shallow layer feature fusion module that combines features from multiple stages of the network and improves scale invariancy and feature representation.

These contributions are collectively aiming to improve feature representation of small objects at the prediction layer that eventually leads to overall better small object detection accuracy. The rest of the paper is organized as follows: the proposed network is described in detail in Section 2, followed by Section 3, which discusses its implementation, evaluation, and results. A discussion on the proposed model is provided in Section 4 followed by the conclusion.

## 2. System Overview

**Figure 3:**
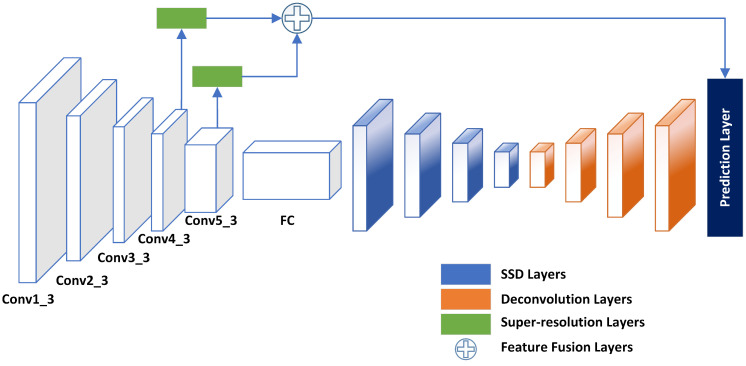
Network architecture of the proposed object detection model. The SSD model is used as the baseline network and extended to include deconvolution module (orange), super-resolution module (green), and shallow layer feature fusion module (**+**).

The proposed small object detection model consists of SSD as the baseline paired with three other modules: a *Shallow Layer Feature Fusion* module, a *Deconvolutional Module*, and a *Super-resolution* module. This model is partially inspired by the Deconvolutional Single Shot Detector (DSSD) [31], Deep CNN with Skip Connection and Network in Network (DCSCN) [51], and Super-Resolution Generative Adversarial Network (SRGAN) [52], and incorporates some variant of these techniques to improve small object detection accuracy. Figure 3 shows the architecture of the proposed small object detector model. In this figure, the super-resolution module along with the feature fusion module are illustrated in green and with a ’+’ symbol, respectively. The SSD layers are illustrated in blue followed by a series of a deconvolution layers (red) that end with a prediction layer.

**Figure 4:**
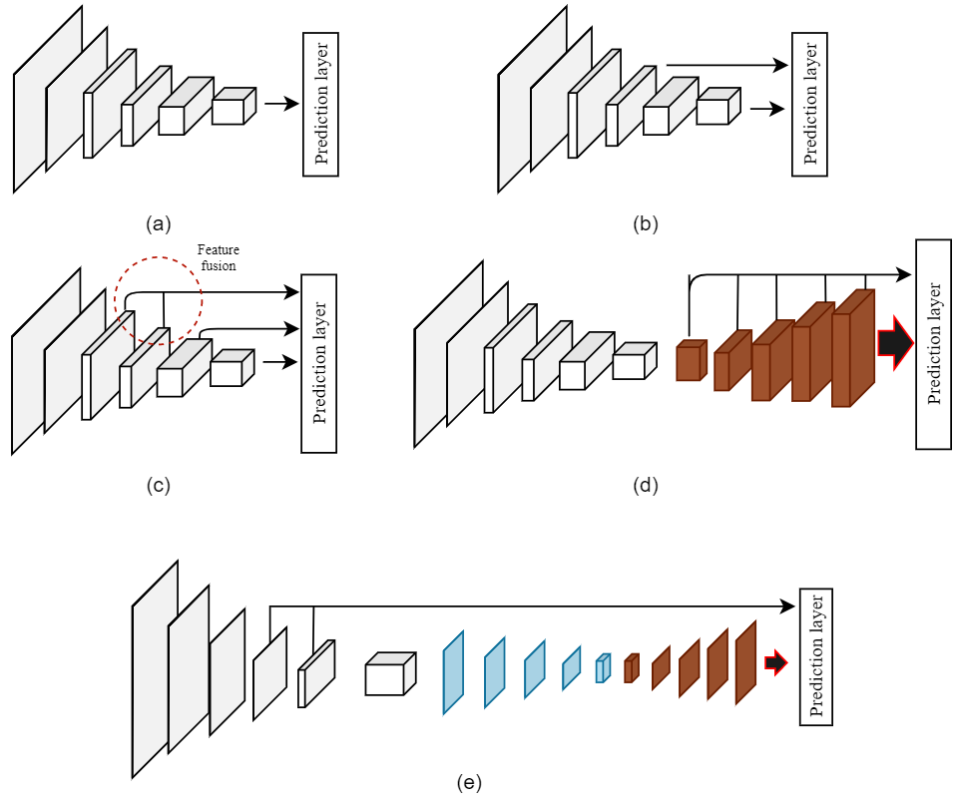
Schematic comparison of approaches used by different types of object detectors. (**a**) Single stage detectors (e.g., YOLO). (**b**) Multi-level features used at prediction layer (e.g., SSD). (**c**) Approach of supplying shallow layer features for prediction (e.g., FSSD). (**d**) Deconvolutional layers for improved feature representation (e.g., DSSD). (**e**) Network schema of our proposed approach.

Figure 4 shows a schematic comparison of the proposed approaches against various other object detectors. Figure 4a represents the approach used by single stage detectors (e.g., YOLO) that solely relies on the final feature representation for detection. These approaches work with relatively limited features that negatively impact small object detection; however, they demonstrate high detection speeds. Figure 4b shows the multi-level presentation of the features at the detection layer that are used by models such as the SSD. They usually perform better for small objects but have relatively lower detection speeds. The shallow layer feature fusion approach shown in Figure 4c and used in models such as the FSSD and the FFSSD improves on the SSD by concatenating additional features from lower layers to enhance small object detection performance. The DSSD approach extends the SSD model by combining the SSD layers with additional deconvolutional layers for better representation of features at the prediction layer, as shown in Figure 4d. Figure 4e shows the structure of our proposed network. The presented approach combines deconvolution and feature fusion methods to provide richer, multi-scale feature maps at the prediction layer. The overall goal of this architecture is to provide a mechanism to collate features from multiple layers across the network and present them at the prediction layer. The multi-scale features at the prediction layer provide enhanced feature representations for small objects and improve their detection accuracy.

### 2.1. Single Shot Multibox Detector (SSD)

The SSD is used as the baseline network in our model due to its high speed and accuracy in object detection. The SSD detector itself is composed of a base network (VGG16) followed by six extra convolutional layers and a Non-Maximum Suppression (NMS) layer for final detection [53]. The base VGG16 network (without its final classification layers) is purely used for feature extraction. The additional six convolutional layers that are attached to the end of the VGG network (apart from the first one) will be used for prediction of the bounding boxes and confidence score for different objects. These layers progressively decrease in their size to accommodate detection of objects at multiple scales; however, due to structural limitation of SSD, small object detection accuracy remains undesirable [27,29].

The SSD’s performance relies heavily on default boxes, specifically their scale and aspect ratios. Each feature map corresponds to a specific scale of default box along with a list of five aspect ratios for each scale. The minimum and maximum scales are set to 0.2 and 0.9, respectively, while aspect ratios are 1, 2, 3, 1/2, and 1/3. The prediction layers receive several feature maps from *“Extra feature layers“*, representing multiple scales/aspect ratios and determining classification scores and bounding box coordinates [16]. The pyramidal feature hierarchy in *“Extra feature layers“* enables SSD detect objects of various sizes in the images; however, its performance for small objects is sub-optimal [27,28,29].

In our proposed model, we extend the multi-scale feature representation concept for enhanced feature representation at the prediction layer by applying the feature fusion, deconvolutional. and super-resolution modules.

### 2.2. Deconvolution Module

In the SSD model after the VGG16 network, the feature maps are scaled down considerably and lack fine details of the objects. The large objects might be sufficiently represented and detected; however, the feature resolution might be insufficient for small objects, leading to poor detection. A deconvolution operation up-scaling the feature resolution [54] and provides more details to the prediction layer. In our proposed network, after the SSD layers, a series of deconvolution layers are added. This structure inspired by [31] partially addresses the inefficiency of feature resolution for small object detection.

**Figure 5:**
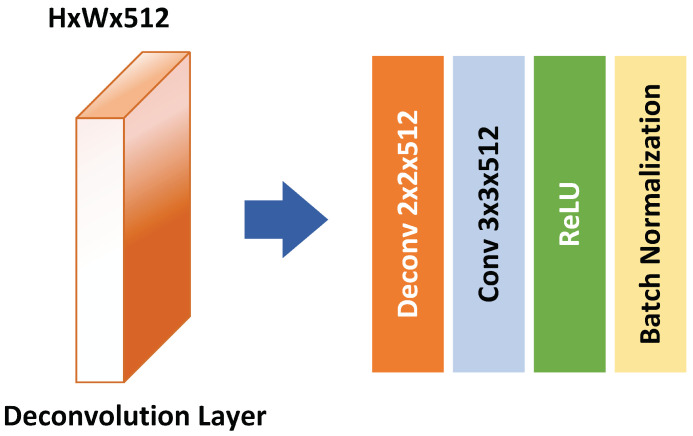
A deconvolutional module unit used after the SSD layers.

The deconvolution module includes a series of five consecutive deconvolution layer that successively increases the feature maps supplied to the prediction layer. This module includes a 2×2 deconvolutional layer with a stride of 2, followed by a convolutional layer activated by the ReLU activation layer and a batch normalization. Figure 5 shows the deconvolution module used in our network. Furthermore, every deconvolution layer is fused with a corresponding SSD of the same resolution size. The fusion is done using an element-wise sum operation followed by a ReLU activation.

**Figure 6:**
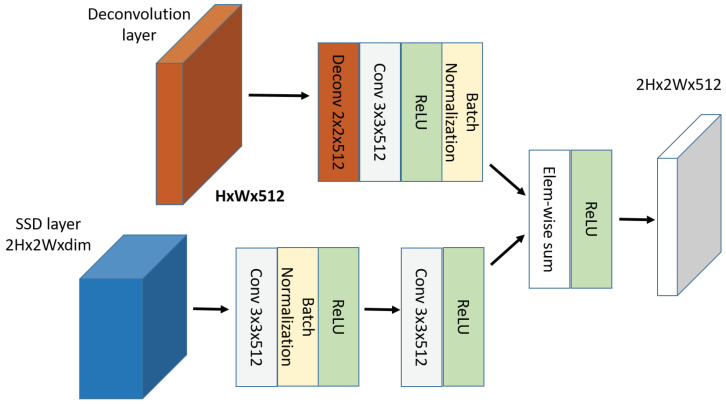
A deconvolutional module unit merged with an SSD layer using element-wise sum operation.

A deconvolutional layer is added for each SSD convolutional layer, effectively up-scaling all the feature layers to be used by the prediction layer. The feature maps of the SSD convolutional layer and deconvolutional layer are combined through an element-wise sum operation. Each SSD layer undergoes two sets of two convolutional operations followed by batch normalization and a ReLU activation layer before combining with the deconvolution feature maps. The element-wise sum operation allows point to point combination of the feature maps at different levels into equivalent weights, as shown in Figure 6.

### 2.3. Super-Resolution Module

The super-resolution (SR) method enables deriving high resolution (HR) features from low resolution (LR) feature maps [55]. SR is a well-explored topic and is applied in various areas, including remote sensing [56] and video SR [57]. In our proposed model, the two shallow layers Conv4_3 and Conv5_3 are considered as LR feature maps. The SR method is applied to derive a HR version of the two shallow layers. Inspired by the techniques proposed by [58,59], we apply an SR technique on the Conv4_3 and Conv5_3 layers to obtain HR feature maps.

**Figure 7:**
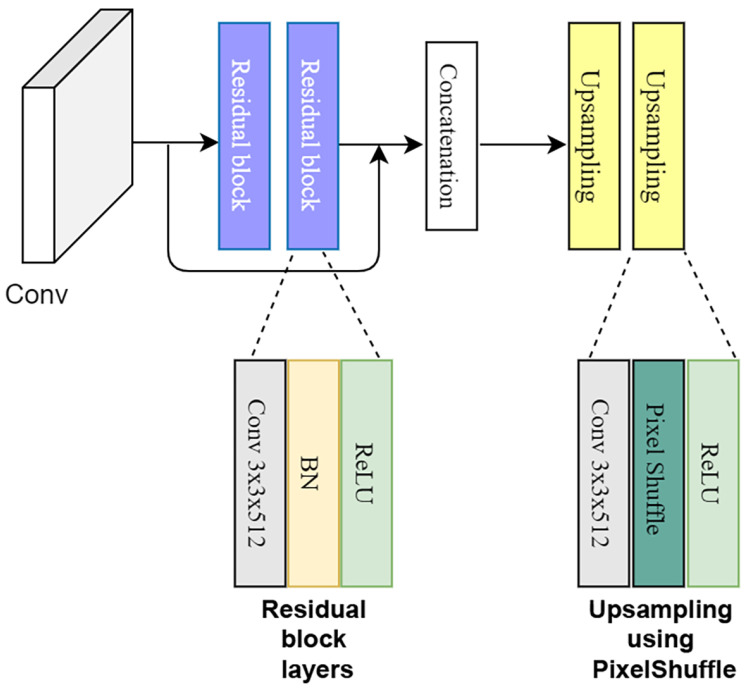
The super-resolution module.

The SR module in our network is based on *residual* and *up-sampling* blocks, as illustrated in Figure 7. Two residual blocks are applied to both of the shallow layers. Furthermore, each residual block consists of two sub-block units of a 3×3 convolutional layer, a batch normalization layer and a ReLU activation layer. As with residual blocks, skip connections are achieved using an elementwise sum operation. The two residual blocks are followed by an elementwise sum operation, concatenating the shallow layer output with the residual layer outputs. The residual blocks enable to obtain multi-scaled features of both shallow layers. The concatenation operation is followed by two up-sampling blocks. The up-sampling blocks help in achieving SR in our model. For the up-sampling purpose, the Pixelshuffle [58] layer, which is used in several SR approaches including [52,59], was employed in this study.

PixelShuffle is an operation used in SR models to increase the spatial resolution of the feature maps. This technique utilizes sub-pixel convolutions with a fractional stride of 1/r (up-sampling ratio) in the LR space. PixelShuffle specifically rearranges elements in a tensor of shape $\left(*,C\times r^{2},H,W\right)$ to a tensor of shape (∗,C,H×r,W×r), where *r* is the up-sampling ratio and *C* is the color channels; essentially, it trades layer depth with higher spatial resolution [58]. The up-sampling block in our proposed architecture consists of a 3×3 convolutional layer, a pixelshuffle layer, and a ReLU activation layer.

### 2.4. Shallow Layer Feature Fusion Module

Multi-scale feature representation works by combining features from multiple stages of the network to provide an enhanced feature map at the prediction layer and improve small object detection accuracy. Such approach has been used in Feature fusion Single Shot multi-box Detector (FSSD) [60], Deconvolutional SSD (DSSD) [31], Feature-Fused SSD (FF-SSD) [61], and Feature fusion and Scaling-based SSD (FS-SSD) [50].

**Figure 8:**
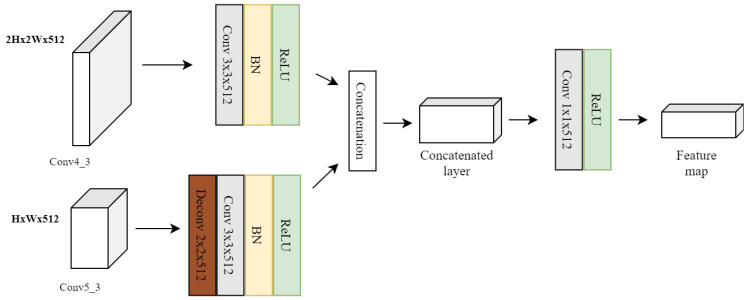
Concatenation of the Conv4_3 and Conv5_3 feature layers.

The feature fusion approach used in our implementation mitigates loss of small object features. The feature maps from the Conv4_3 and Conv5_3 are fused and supplied to the prediction layer. There are two different ways to fuse features from shallow layers: *concatenation* and *element-wise summation*. The concatenation approach requires inputs with matching shapes except for the concatenation axis. However, element-wise summation works with tensors with similar batch sizes and follows arrays broadcasting rules [62]. In the shallow layer feature fusion module, a 1×1 convolution layer is applied after the concatenation, which leads to better learning and enhancement of small objects’ features over the background. The concatenation operation is illustrated in Figure 8.

The Conv5_3 layer feature resolution differs from the resolution of the Conv4_3 feature layer. For fusion of both the layers, the Conv5_3 layer is up-sampled to the same size of the Conv4_3 layer. The up-sampling is achieved through a deconvolution operation at the Conv5_3 layer. Next, both the feature layers undergo a 3×3 convolution operation, batch normalization, and ReLu activation function. The output feature map of the feature fusion module is supplied to the prediction layer. In comparison to the standard SSD network, the prediction layer of our model receives more feature maps with potentially better representation of the small objects.

## 3. Experiments

The proposed model is evaluated for its object detection accuracy with an emphasis on small object detection. The details of multiple experiments conducted with the proposed model are presented in the following sections.

### 3.1. Datasets

**Figure 9:**
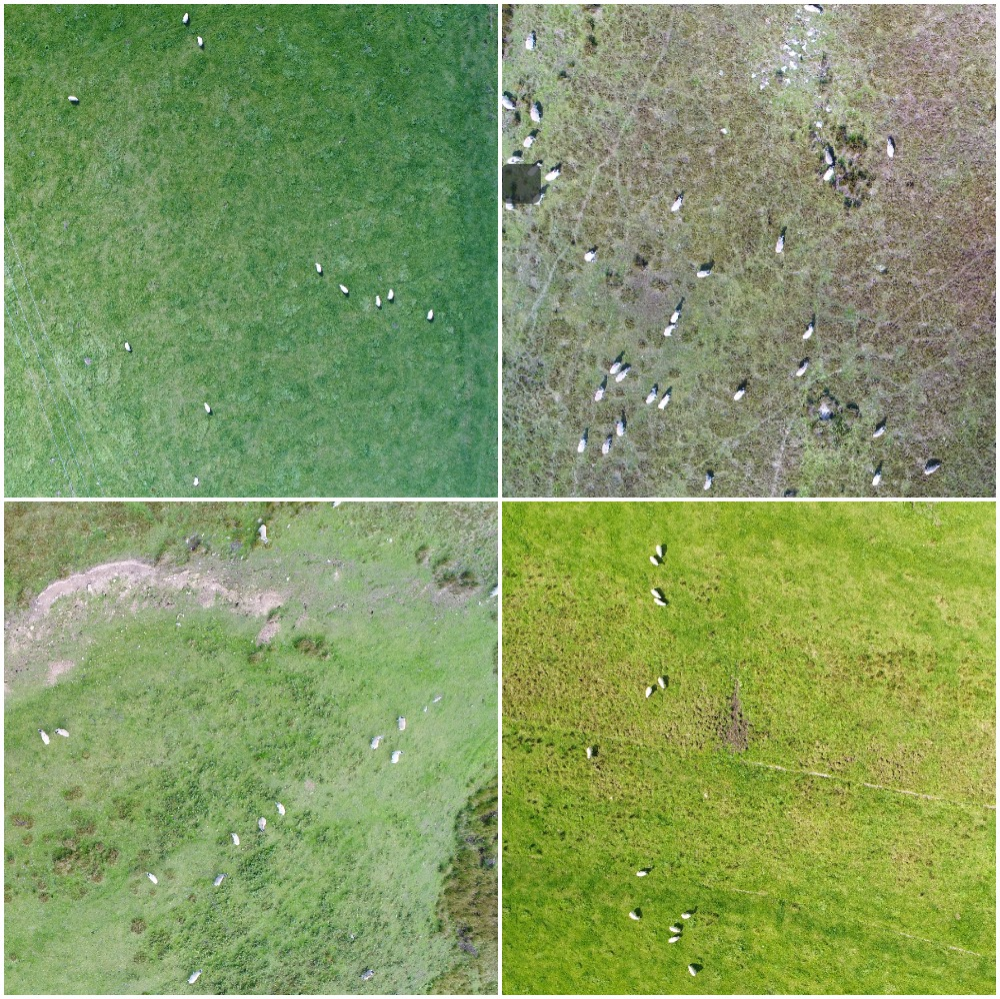
Example images of the livestock dataset. In the images, sheep are small targets for the object detectors.

The model was evaluated on three datasets: a custom UAV image dataset of livestock captured as part of the 5G rural integrated test-bed (5GRIT) project [63], the Stanford Drone Dataset (SDD) [11], and a crowd monitoring dataset acquired as part of the European Union (EU) project, MONICA [64]. The custom livestock dataset consists of aerial images of livestock captured over farms across the UK. The dataset includes only one labeled class of livestock (sheep). The images were captured from a UAV flown at 50 m altitude. In total, 425 RGB images with a very high resolution of 5400×3600 pixels were acquired. Figure 9 shows some example images of the dataset.

As can be seen in Figure 9, the livestock dataset exhibits aerial images of livestock (sheep) with considerably small spatial size proportional to the total image size. This poses a challenge to the existing state-of-the-art object detectors, which make this dataset a suitable test-bed and use case to evaluate our small object detector performance. Since images in the livestock dataset have very high resolution, it is not possible to use them directly for the training of a neural network model. Hence, each image was split (cropped) into multiple images of 300×300 resolution. As a result of this operation, a total of 3900 images were available for training based on the livestock dataset.

The SDD dataset consists of 400 aerial images of people captured using a UAV at an original resolution of 6000×4000 pixels. The *Pedestrian* category images were used for training and evaluation in our study. Since images in the SDD dataset are of very high resolution, each image was split into multiple images of 600×400 for training. We used different cropping factors in livestock (300×300) and the SDD dataset (600×400) mainly to equalize the average scale of small objects across both datasets. Livestock dataset objects (sheep) are considerably smaller than benchmark SDD objects (Pedestrian). This helped to improve the consistency of our experiments and results across both datasets. Figure 1 shows some example images of this dataset. After training and evaluation using the SDD dataset on *Pedestrian* category, the MONICA dataset, which consists of images of the crowd, is used for further evaluation of our proposed model.

### 3.2. Implementation

The proposed model is based on the SSD object detector. For our experiments, the Keras implementation of SSD with VGG16 used in [65] is employed as our baseline architecture network. For training purposes, parameters from the original SSD implementation is used in our studies. The anchor box ratios, scaling factors, and other parameters were kept unchanged from the original recommendation. To improve the volume of the training dataset, the data augmentation process recommended in the original SSD implementation is adopted to increase the training dataset image count. The data augmentation process includes randomly sampling an entire original input image such that it has a minimum Jaccard index with objects of around 0.1, 0.3, 0.5, 0.7, or 0.9, and then randomly sampling a patch. The batch size was set to 32 for training and the learning rate ranged from 0.001 for the first 60 k steps, 0.0001 for up to 80 k and 0.00001 for 100 k steps. The maximum iteration was set to 100 k steps.

### 3.3. Comparison on the Livestock Dataset

The performance of our model is compared with several popular CNN-based object detectors including Faster R-CNN [20], variants of SSD including SSD300 and SSD512 [16], YOLOv3 [66], and CenterNet++ [30]. Moreover, the proposed model has been compared with several state-of-the-art small object detectors including Deconvolutional SSD (DSSD) [31], FS-SSD [50], FF-SSD [61], MPFPN [49], and EFPN [46].

The mean Average Precision (mAP) of all the detectors are shown in Table 2. The mAP is adopted as the primary criterion (Figure of merit) for detection accuracy, which is an indicator related to the Intersection over Union (IoU) threshold. We take the most used threshold IoU = 0.5 in our experiments.

All the target objects in the Livestock dataset have been checked for fulfillment of DORI’s small object criterion [25]. Bear in mind that, objects in the Livestock dataset are considerably small (significantly smaller than what DORI outlines as small object) and general purpose object detectors in this comparison such as YOLOv3, SSD, CenterNet++, and Faster R-CNN are not purely made to deal with such small scale of objects. The enclosure of general purpose object detectors in this comparison is mainly to demonstrate how purposely made small object detectors can positively contribute to the small object detection performance in aerial imagery.

For the livestock dataset, our proposed model achieves the highest accuracy with a mAP of 79.12%. The YOLOv3 and Faster R-CNN show the lowest mAP and Recall compared to other detectors considered in the experiment. Among general purpose object detectors, CenterNet++ with a respective mAP and Recall of 76.18 and 92.44 ourperformed other general purpose object detector; however, it came short when compared to small object detectors. In terms of Recall, once again the proposed model, with 94.10%, outperformed all other detectors in this comparison. The FS-SSD, with a mAP and Recall of 77.14% and 93.91%, respectively, was the second-best detector in this comparison; however, with 17.35 Frames per Second (FPS), FS-SSD outperformed our model in this respect.

**Figure 10:**
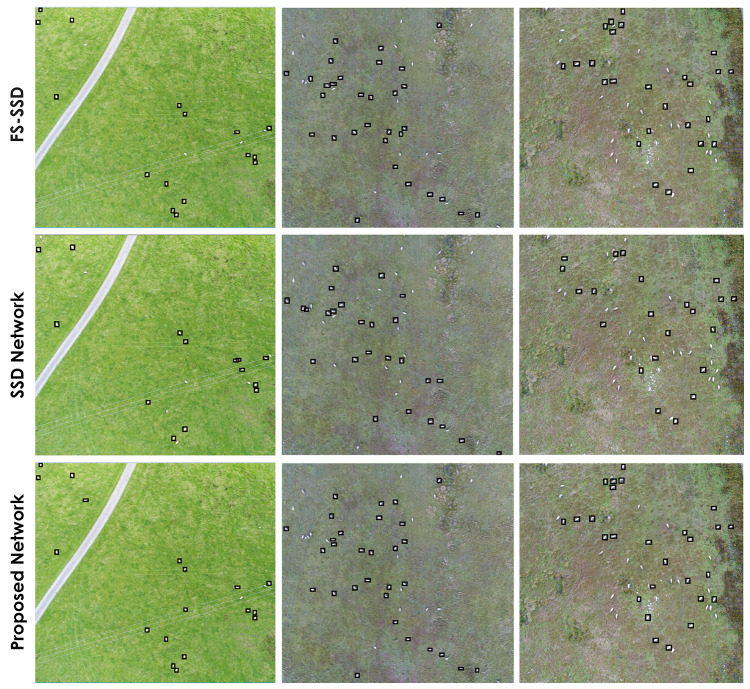
Comparison of small object detection between the proposed network (**bottom row**), the SSD network (**middle row**) and the FS-SSD network (**top row**) on the custom livestock dataset.

Small object detection is important in UAV and satellite imagery as the object sizes are usually small relative to the total image size. The livestock dataset consists of considerably small top-down images of livestock (sheep) and our proposed model performs well in detecting these objects compared to other methods in this comparison. Figure 10 shows a qualitative comparison of the original SSD300 object detector, FS-SSD, and our proposed model. Images in Figure 10 have been chosen randomly to retain the fairness of our qualitative comparison. It can be observed that the SSD misses several instances of livestock across the images; however, FS-SSD’s performance and accuracy is very comparable to our proposed model. It is worth mentioning that due to extremely small object sizes, all models in this comparison fail to detect sheep in a few instances. Keep in mind that the majority of the models in this comparison are very close in terms of mAP and Recall values (around 2% mAP difference between our model and the second best model in this study based on livestock dataset results) and qualitative comparison of these models using a few random sample images might not be a good indication of their overall performance.

**Figure 11:**
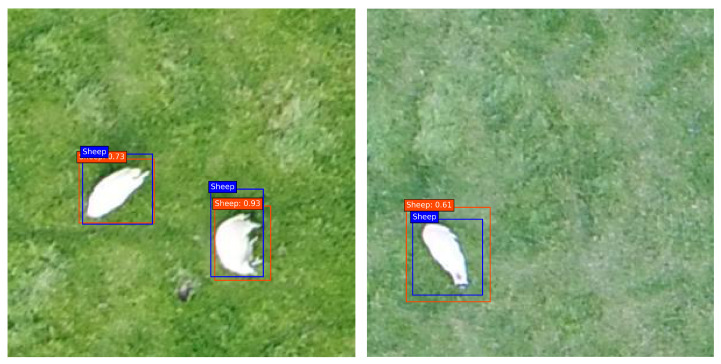
Qualitative evaluation of bounding box predictions by the proposed network on custom livestock dataset. Blue boxes correspond to the ground truth label and red boxes are the predicted bounding boxes.

Furthermore, a qualitative evaluation of the proposed model detection performance is shown in Figure 11. The ground truth and the predicted bounding boxes are shown in blue and red, respectively. It can be observed that the prediction bounding boxes are reasonably aligned with the ground truth and the object in the image.

### 3.4. Comparison with the Stanford Drone Dataset (SDD)

One major use case for small object detection is person detection and localization from aerial images. Hence, we attempted to evaluate and compare our model performance in this area. For this experiment, the *Pedestrian* category images from the Stanford Drone Dataset (SDD) is considered [11]. This dataset has been used by many other researchers for small object detection in aerial imagery. Contenders of this comparison are the same as our previous comparison on the Livestock dataset.

The quantitative results of this comparison are shown in Table 3. In terms of *Pedestrian* detection, our proposed model achieves the highest mAP of 68.71%, followed closely by DSSD and FS-SSD with a mAP of 66.20% and 66.02% respectively. Again, general purpose object detectors including YOLOv3 and Faster R-CNN show the lowest mAP when compared to other detectors in the experiment. Among general purpose object detectors only, once again, CenterNet++, with respective a mAP and Recall of 66.01 and 83.91, performed the best; however, it came short when compared to small object detectors such as the proposed, DSSD, and FS-SSD. In terms of Recall, DSSD with 87.26% outperformed any other models in this comparison including our proposed model, with a Recall rate of 85.95%. In terms of inference speed, our proposed model with an average FPS of 8.75 meets the requirements of real-time detection. However, for applications such as oncoming traffic analysis, where detection speed is critical, FF-SSD with 42.51 FPS might be the preferred option.

**Figure 12:**
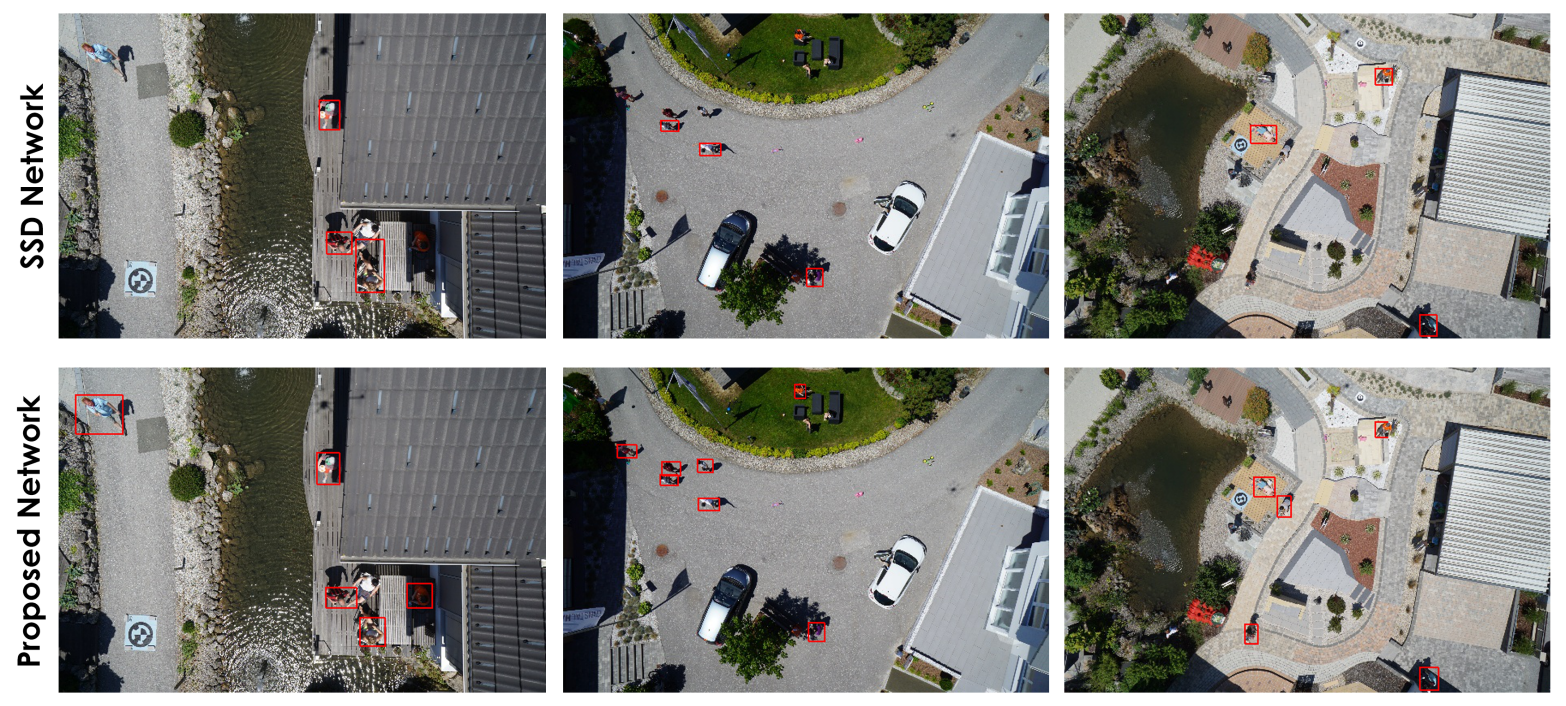
Comparison of small object detection of our proposed network (bottom row) with the SSD network (top row) onthe *Pedestrian* category from the Stanford drone dataset.

Figure 12 shows a qualitative comparison of the proposed model against SSD300 using sample images from the SDD dataset. It can be observed that the SSD300 misses several instances of Pedestrian across the sample images. However, as the models in this comparison are competing closely in terms of mAP and Recall values, qualitative comparison using a few random sample images might not be a good indication of their overall performance.

**Figure 13:**
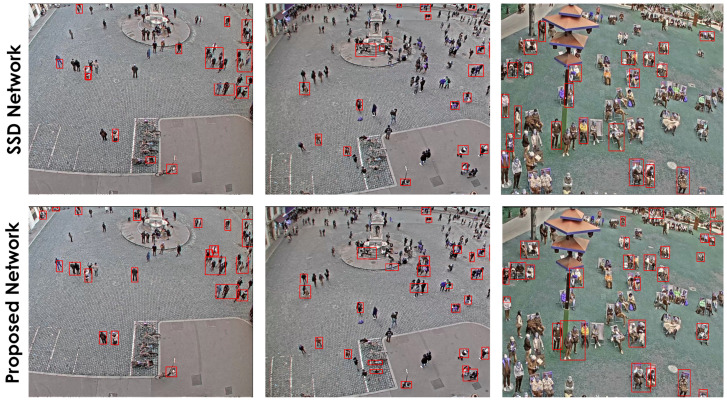
Comparison of small object detection of our proposed network (**bottom row**) with SSD network (**top row**) on a custom dataset acquired under MONICA project data.

To identify how our model performs in other similar datasets, the trained model on the SDD dataset has been tested qualitatively for person detection on images obtained from the MONICA project dataset [64]. Images in the MONICA dataset were captured from surveillance cameras at various public outdoor events. Due to an insufficient number of labeled samples in this dataset, we have only reported the qualitative comparison results mainly to demonstrate how these models perform under different conditions and they can be adopted to different scenarios and circumstances. The qualitative results for person detection using the MONICA dataset are shown in Figure 13, while both models exhibit inferior results in terms of Recall compared to what we saw in the previous experiments, the proposed model seems to have a slight edge over the SSD in this comparison. A drop in both models’ performance was predictable, as these models were trained on a different dataset (SDD) with a relatively different nature, camera perspective, object scale, and lighting condition. Although this might be less than ideal, it shows the adaptability of the proposed model to similar real-world scenarios such as surveillance. Due to a limited number of labeled images in the MONICA dataset, we are unable to provide statistically reliable quantitative comparison results. Furthermore, on some occasions, the proposed model mistakenly combines the bounding boxes of the nearby objects. We believe this is mainly caused by the difference in the objects’ scale in training (SDD) and testing (MONICA) datasets and can be mitigated by readjusting the raw input image slicing-factor (cropping) to equalize the average size of objects in the training dataset (SDD) and testing datasets (MONICA).

### 3.5. Ablation Studies of the Proposed Network

Ablation studies allow us to identify the impact of different modules on our model performance and speed. These experiments were conducted incrementally in isolation to identify how feature fusion, super-resolution, and deconvolution modules individually and collectively improve baseline SDD300 model performance and how they impact its inference speed in term of FPS in small object detection. The ablation study is conducted on the livestock dataset. The results of the ablation study are shown in Table 4. In the ablation study, SSD300 in the void of other modules is considered as the baseline network of our proposed model and achieved mAP 74.80%. The first study is to evaluate the shallow layer feature fusion model, wherein both the *element-wise sum* and the *concatenation* operations are evaluated. The *element-wise sum* method improved the accuracy of SSD300 from 74.80% to 75.70%. However, the *concatenation* method of feature fusion showed a slightly better performance of 76.10%. Hence, for the remaining ablation studies of the *deconvolutional* and the *super-resolution* modules, the *concatenation* feature fusion approach used as the preferred option. In terms of FPS, there is no significant difference between the *element-wise sum* and the *concatenation* operations and both of these techniques drop the FPS from 36.50 in the baseline SSD300 to around 22 FPS. Next, we attempted to identify how the shallow layer feature fusion (concatenation) performs along with the super-resolution module. The result shows improved performance of 77.20% was achieved as a result of this combination. In terms of FPS, inclusion of super-resolution module dropped the FPS from 22.42 to 17.64. The ablation study on the combination of deconvolutional and the shallow layer feature fusion (concatenation) further improved the mAP to 77.90%, but it dropped the inference FPS down to 14.52 only. Finally, the complete proposed model, including the super-resolution, the shallow layer feature fusion (concatenation), and the deconvolutional module, attained the best mAP performance of 79.12%; however, it slowed down the baseline SSD300 from 36.50 to 8.75 FPS.

## 4. Discussion

UAV imagery is getting more popular than ever before with a variety of applications, including urban planning, smart farming, search and rescue, and security and surveillance. Due to many intrinsic characteristics of UAV imagery, such as high flight altitude, top-down camera perspective, and wide-angle lenses, objects in aerial imagery appear to have a distinctive shape and spatial properties and they usually take up a small fraction of the entire frame, which may pose a challenge to conventional object detectors.

**Figure 14:**
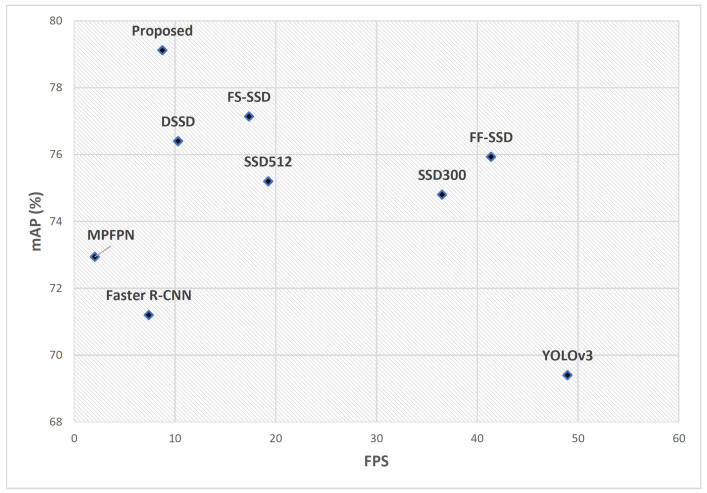
Speed and accuracy comparison of the proposed method with the state-of-the-art methods on the livestock dataset. It can be observed that the proposed model supersedes other approaches in terms of mAP, which makes it suitable for applications where accuracy is critically important.

Many researchers have attempted to address small object detection by introducing additional feature enhancement modules to conventional object detectors. Likewise, the proposed model in this research utilizes the SSD as the baseline network and improves its small object detection performance by incorporating deconvolution, super-resolution, and feature fusion modules. These modifications allow better feature representation of small objects at the prediction layer, which improves the baseline model’s mAP (IoU = 0.5) in small object detection while retaining the requirements for the majority of real-time applications. Figure 14 shows the speed and accuracy comparison of the proposed model against the state-of-the-art models on the livestock dataset.

**Figure 15:**
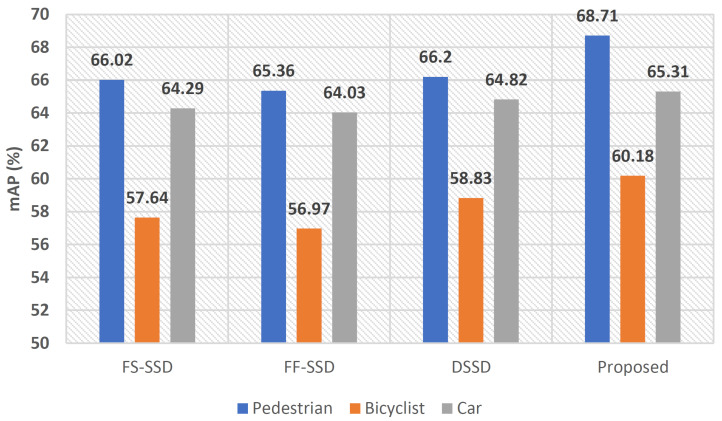
Comparison between the proposed model mAP with some other object detectors on the SDD dataset. Car, Bicyclist, and Pedestrian categories were considered in this comparison.

As can be observed in Figure 14, the proposed model trumped other models in this comparison in terms of mAP. Other models, such as FS-SSD and FF-SSD, also managed to deliver reasonably good accuracy; however, their superior performance in terms of FPS makes them a more desirable option for applications such as oncoming traffic analysis where detection speed is critical. The proposed model can be used in applications such as precision farming, search and rescue, and disaster management, where accuracy is critically important. The majority of the images in the livestock dataset exhibit a low-contrast scenery of greenery with distinct foreground objects (livestock), which might skew the detection results. Hence, besides the livestock dataset, we have tested our model performance on the Stanford Drone Dataset (SDD). The SDD is a popular benchmark dataset used by many researchers [11,50,67,68] for (small) object detection in aerial imagery. Again, our comparative results showed the superiority of the proposed model over methods in the comparison. Although the focus of our comparison was mainly on the *Pedestrian* category of the SDD dataset, further investigations showed that our model performs equally well in some other object categories, including *Bicyclist* and *Car* on the SDD dataset. Figure 15 illustrates this comparison.

**Figure 16:**
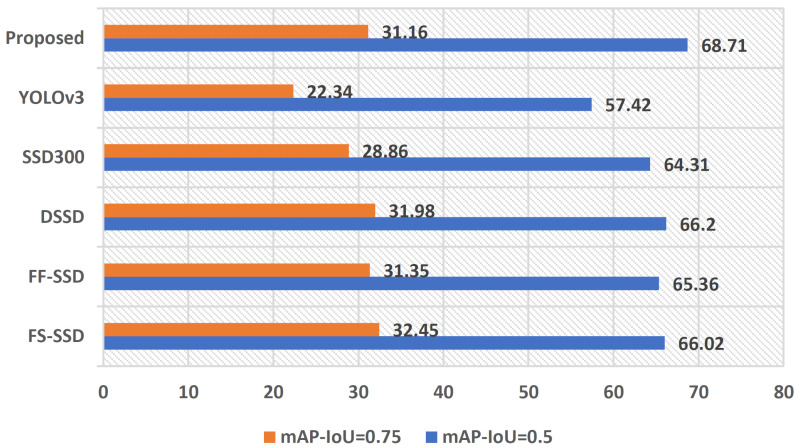
Comparison of mAP with IoU of 0.5 and 0.75 on the *Pedestrian* category of the SDD dataset.

Beside the mean average precision (mAP) with an IoU of 0.5, we have attempted to investigate how the proposed model performs and compares with other detectors using a challenging IoU of 0.75. As expected, we observed a significant drop in mAP across all models involved in the comparison. Although our model is not the best performer in this experiment, it performs comparably with other detectors in this study and is better than the baseline model. Figure 16 illustrates comparison of mAP with IoU of 0.5 and 0.75 on *Pedestrian* category of SDD dataset across selected models in this study. Such an abrupt drop in accuracy indicates models in this comparison are having a hard time aligning the bounding boxes with the ground-truth labels in the regression process. Due to the smaller size of the objects, a slight misalignment significantly degrades the area of overlap and increases the area of union, which negatively impacts mAP.

The results of our experiments and ablation studies imply deconvolution, super-resolution, and feature fusion modules enhanced feature representation of small objects at the prediction layer, which results in better accuracy in small object detection. Our proposed model not only improved the overall accuracy of the baseline SSD300 model but also competes with some of the state-of-the-art small object detectors.

## 5. Conclusions

In this paper, an object detection model with the goal of improving small object detection in aerial images is presented. The proposed model extends the SSD using the methods of deconvolution, super-resolution, and shallow layer feature fusion. The proposed extension enhances the feature representation of the objects at the prediction layer and leads to improved object detection accuracy. This approach is particularly beneficial for small object detection because with many state-of-the-art object detectors, the features of small objects are not represented sufficiently at the prediction layer for a reliable detection. The proposed model was trained and evaluated on two datasets: a custom UAV dataset of livestock, and the Stanford drone dataset. The results of the experiments showed that the proposed model performs mostly better than other object detectors considered in the comparative study. The proposed model can be used in applications such as precision farming, search and rescue, and disaster management, where accuracy is critically important.

## Figures and Tables

**Table 1:** A summary of selected works on CNN-object detection for small objects in images.

Strategy	Authors	Model Features	Data	Results
Two-stage detectors	[32]	Feature extraction CNN combined with the R-CNN framework	Mobile Mapping Systems (MMS) images	mAP of up to 85%. Comparatively, 12% higher accuracy than ResNet-152
	[33]	R-CNN network combined with Tiny-Net, global attention block followed by a final classification block	Remote sensing images	Higher detection accuracy than R-CNN variants
	[34]	A R-CNN network combined with a deconvolution layer	Remote sensing images	Higher accuracy than Faster R-CNN is reported
	[35]	A region proposal network combined with fusion network that concatenates spatial and semantic information	Remote sensing	Improved detection accuracy compared to state-of-the-art
	[27]	Multi-block SSD consists of three stages, including patching, detection, and stitching	Railway scene dataset	Improved detection rate of small objects by 23.2% in comparison with the baseline object detectors
Single stage detectors	[36]	Various configurations of SSD architecture, including stride elimination at different parts of the network	MS COCO dataset	Better detection accuracy for small objects in the COCO dataset when compared to baseline SSD
	[25]	Tiling-based approach for training and inference on an SSD network	Micro aerial vehicle imagery	Improved the detection performance on small objects when compared with full frame approaches
	[37]	Modification of YOLOv3 model for multi-scale feature representation	UAV imagery	Improvement in small object detection when compared to base YOLOv3 model
	[38]	YOLO model with multi-scale feature fusion	Traffic imagery for car accident detection	Able to detect car accidents in 0.04 seconds with 90% accuracy
	[39]	Feature fusion and feature dilation combined with YOLO model	Vehicle imagery	Improved accuracy in the range of 80% and 88% on different datasets
	[40]	YOLOv3 Residual blocks optimized by concatenating two ResNet units that have the same width and height	UAV imagery	Improved IoU to over 70% to 80% across different datasets compared with the baseline models
	[41]	Region Context Network attention mechanism shortlists most promising regions, while discarding the rest of the input image to keep high resolution feature maps in deeper layers.	USC-GRAD-STD and MS COCO dataset	Improvement in average precision from 50.8% in baseline models to 57.4%
	[42]	Feature fusion and spatial attention-based Multi-block SSD	LAKE-BOAT dataset	79.3% mean average precision
Super-resolution	[43]	Patch-based and pixel-based CNN architectures for image segmentation to identify small objects	Remote sensing images	Classification accuracy of 87% reported
	[26]	A super-resolution-based generator network for up-sampling small objects	COCO dataset	Improved detection performance on small objects when compared with R-CNN models
	[44]	Super-resolution method for feature enhancement to improve small object detection accuracy	Several RGB image datasets	Better detection accuracy compared to other super-resolution-based methods
	[45]	A super-resolution-based Generative Adversarial Network (GAN) for small object detection	Several RGB image datasets	Achieved higher detection accuracy in comparison to R-CNN variants
Feature Pyramids	[46]	Extended feature pyramid network which employs large-scale super-resolution features with rich regional details to decouple small and medium object detection	Small traffic-sign Tsinghua-Tencent and MS COCO dataset	Better accuracy across both datasets compared to the state-of-the-art methods
	[47]	A two-stage detector (similar to the Faster-RCNN) which first adopts the feature pyramid architecture with lateral connections, then utilizes specialized anchors to detect the small objects from large resolution image	Small traffic-sign Tsinghua-Tencent dataset	Significant accuracy improvement compared with state-of-art methods
	[48]	A parallel feature pyramid network constructed by widening the network width instead of increasing the network depth. Spatial pyramid pooling adopted to generate a pool of feature	MS-COCO dataset	7.8% better average precision over latest variant of SSD
	[49]	Multi-branch parallel feature pyramid network (MPFPN) used to boost feature extraction of the small objects. The parallel branch is designed to recover the features that missed in the deeper layers and a supervised spatial attention module used to suppress background interference	VisDrone-DET dataset	Competitive performance compared with other state-of-the-art memthods
	[50]	Feture fusion and scaling-based SSD network with spatial context analysis	UAV imagery	Achieved 65.84% accuracy on PASCAL Visual Object Classes dataset. High accuracy on small objects in UAV images

**Table 2:** The mAP, Recall, and FPS comparison of the proposed model with the state-of-the-art small object detectors on our custom livestock dataset. Some of the general purpose object detectors have been included in this comparison.

Model	FPS	Recall (%)	mAP (%)
SSD300	36.50	88.20	74.80
SSD512	19.25	91.32	75.20
CenterNet++	4.70	92.44	76.18
YOLOv3	**48.95**	78.23	69.40
Faster R-CNN	7.40	83.60	71.20
DSSD	10.30	93.15	76.40
FS-SSD	17.35	93.91	77.14
FF-SSD	41.36	91.01	75.93
MPFPN	2.04	86.18	72.94
EFPN	4.14	90.23	74.81
**Proposed**	8.75	**94.10**	**79.12**

**Table 3:** The mAP, Recall, and FPS comparison of the proposed model with state-of-the-art small object detectors on a subset of SDD dataset containing aerial images. Some of the general purpose object detectors have been included in this comparison.

Model	FPS	Recall (%)	mAP (%)
SSD300	36.40	81.45	64.31
SSD512	19.35	83.58	65.24
CenterNet++	4.72	83.91	66.01
YOLOv3	**49.20**	78.64	57.42
Faster R-CNN	7.40	80.75	59.60
DSSD	10.30	**87.26**	66.20
FS-SSD	18.05	85.88	66.02
FF-SSD	42.51	83.66	65.36
MPFPN	2.35	79.32	61.79
EFPN	4.33	82.11	63.94
**Proposed**	8.75	85.95	**68.71**

**Table 4:** Ablation study of the proposed network on the livestock dataset. Different combinations of the feature fusion methods, super-resolution, and deconvolution were evaluated based on mAP and FPS.

Feature Fusion	Deconvolution	SuperResolution	mAP	FPS
NA	NA	NA	74.80	36.50
Element-wise sum	NA	NA	75.70	22.86
Concatenation	NA	NA	76.10	22.42
Concatenation	NA	YES	77.20	17.64
Concatenation	YES	NA	77.90	14.52
Concatenation	YES	YES	79.12	8.75

## Data Availability

Data and source code can be made available upon request from the corresponding author.
